# L-Type Ca^2+^ Channel Sparklets Revealed by TIRF Microscopy in Mouse Urinary Bladder Smooth Muscle

**DOI:** 10.1371/journal.pone.0093803

**Published:** 2014-04-03

**Authors:** Peter Sidaway, Noriyoshi Teramoto

**Affiliations:** Department of Pharmacology, Faculty of Medicine, Saga University, Saga City, Japan; Cinvestav-IPN, Mexico

## Abstract

Calcium is a ubiquitous second messenger in urinary bladder smooth muscle (UBSM). In this study, small discrete elevations of intracellular Ca^2+^, referred to as Ca^2+^ sparklets have been detected in an intact detrusor smooth muscle electrical syncytium using a TIRF microscopy Ca^2+^ imaging approach. Sparklets were virtually abolished by the removal of extracellular Ca^2+^ (0.035±0.01 vs. 0.23±0.07 Hz/mm^2^; *P*<0.05). Co-loading of smooth muscle strips with the slow Ca^2+^ chelator EGTA-AM (10 mM) confirmed that Ca^2+^ sparklets are restricted to the cell membrane. Ca^2+^ sparklets were inhibited by the calcium channel inhibitors R-(+)-Bay K 8644 (1 μM) (0.034±0.02 vs. 0.21±0.08 Hz/mm^2^; *P*<0.05), and diltiazem (10 μM) (0.097±0.04 vs. 0.16±0.06 Hz/mm^2^; *P*<0.05). Ca^2+^ sparklets were unaffected by inhibition of P2X_1_ receptors α,β-meATP (10 μM) whilst sparklet frequencies were significantly reduced by atropine (1 μM). Ca^2+^ sparklet frequency was significantly reduced by PKC inhibition with Gö6976 (100 nM) (0.030±0.01 vs. 0.30±0.1 Hz/mm^2^; *P*<0.05), demonstrating that Ca^2+^ sparklets are PKC dependant. In the presence of CPA (10 μM), there was no apparent change in the overall frequency of Ca^2+^ sparklets, although the sparklet frequencies of each UBSM became statistically independent of each other (Spearman's rank correlation 0.2, *P*>0.05), implying that Ca^2+^ store mediated signals regulate Ca^2+^ sparklets. Under control conditions, inhibition of store operated Ca^2+^ entry using ML-9 (100 μM) had no significant effect. Amplitudes of Ca^2+^ sparklets were unaffected by any agonists or antagonists, suggesting that these signals are quantal events arising from activation of a single channel, or complex of channels. The effects of CPA and ML-9 suggest that Ca^2+^ sparklets regulate events in the cell membrane, and contribute to cytosolic and sarcoplasmic Ca^2+^ concentrations.

## Introduction

In smooth muscle, intracellular Ca^2+^ is a ubiquitous second messenger that controls virtually every physiological process including growth, contraction, division, and cell death [1]. At rest, the smooth muscle cells of the detrusor maintain a level of muscle tone that reflects bladder fullness. In order to engage micturition, detrusor smooth muscle (DSM) must then contract forcefully to expel the accumulated urine [2]. To enable contractions to occur, Ca^2+^ enters urinary bladder smooth muscle (UBSM) cells *via* L-type voltage-gated Ca^2+^ channels (VGCCs). During UBSM cell depolarization, several VGCCs are simultaneously activated in different areas of the cell membrane, resulting in Ca^2+^ entry, followed by Ca^2+^-induced Ca^2+^ release (CICR) and a whole-cell Ca^2+^ transient (WCT), which propagates rapidly through gap junctions, allowing micturition to take place [2],[3].

Due to the vital role that intracellular Ca^2+^ plays in many cellular signalling processes, the maintenance of Ca^2+^ homeostasis in both the cytosol (Ca^2+^_cyt_) and the sarcoplasmic reticulum (Ca^2+^_sr_) is of significant importance. Following a WCT, Ca^2+^_cyt_ is rapidly reduced to resting levels by a combination of plasma membrane (PMCA) and sarcolemmal (SERCA) Ca^2+^-ATPase activities [4], Na^+^/Ca^2+^ exchange [5], and mitochondrial Ca^2+^ uptake [6]. The exact contributions of each pathway vary according to the organ studied, age and species [1].

Such is the ubiquitous role of Ca^2+^ signalling in UBSM cells, a range of signals can occur independently of VGCC activation, e.g. Ca^2+^ sparks, puffs and waves, which in UBSM are generated by Ca^2+^ release from the sarcoplasmic reticulum (SR) [7]. The occurrence of SR-dependent Ca^2+^ signals would imply that Ca^2+^_sr_ can become depleted independently of Ca^2+^_cyt_, thus indicating a need for Ca^2+^ entry that promotes store refilling without necessarily activating smooth muscle contraction, an effect originally described as “capacitative Ca^2+^ entry” [8], and currently described as “store-operated Ca^2+^ entry” (SOCE) [9].

The relatively recent, more widespread use of total internal reflection fluorescence (TIRF) microscopy in cellular imaging has revealed the presence of small VGCC-mediated events that are restricted to the membrane of isolated vascular smooth muscle cells [10],[11]. It has been suggested that these events, that occur at RMPs not typically associated with VGCC activation, termed Ca^2+^ sparklets are of significant importance to both local and global intracellular Ca^2+^ concentrations [12], and are apparently unaffected by depletion of Ca^2+^_sr_ [13].

The aim of this research was to investigate the presence of Ca^2+^ sparklets in smooth muscle strips isolated from mouse urinary bladder, using an adapted TIRF microscopy approach. The relationship between Ca^2+^_sr_ and Ca^2+^ sparklets was also investigated.

## Methods

### Ethics statement

Male C57BL/6 mice between 6 and 10 weeks of age were killed by cervical dislocation. Efforts were made to minimise the suffering of experimental animals used in this study. All animal experiments were approved by the animal care and use committee of Saga University (Saga, Japan).

### Dissection and tissue preparation

Urinary bladders were removed from the mice following cervical fracture. Isolated urinary bladders were sustained in an oxygenated Krebs solution, consisting of (in mM): NaCl 118.4, NaHCO_3_ 25.0, NaH_2_PO_4_ 1.13, KCl 4.7, glucose 11.1, CaCl_2_ 1.8, and MgCl_2_ 1.3. To ensure adequate oxygenation and to maintain pH between 7.3–7.4, solutions were bubbled with a mixture of 95% O_2_ and 5% CO_2_ gas. The ventral wall of the urinary bladder was opened longitudinally from the urinary bladder neck (posterior) to the top of the dome (anterior), and pinned to a Sylgard-coated surface. Urothelium was carefully removed from each individual strip. Urinary bladder strips (4–6 mm width and 10–15 mm length) were cut along the craniocaudal axis of the DSM, ensuring that several intact smooth muscle bundles were present in each strip.

### TIRF microscopy

**Figure 1:**
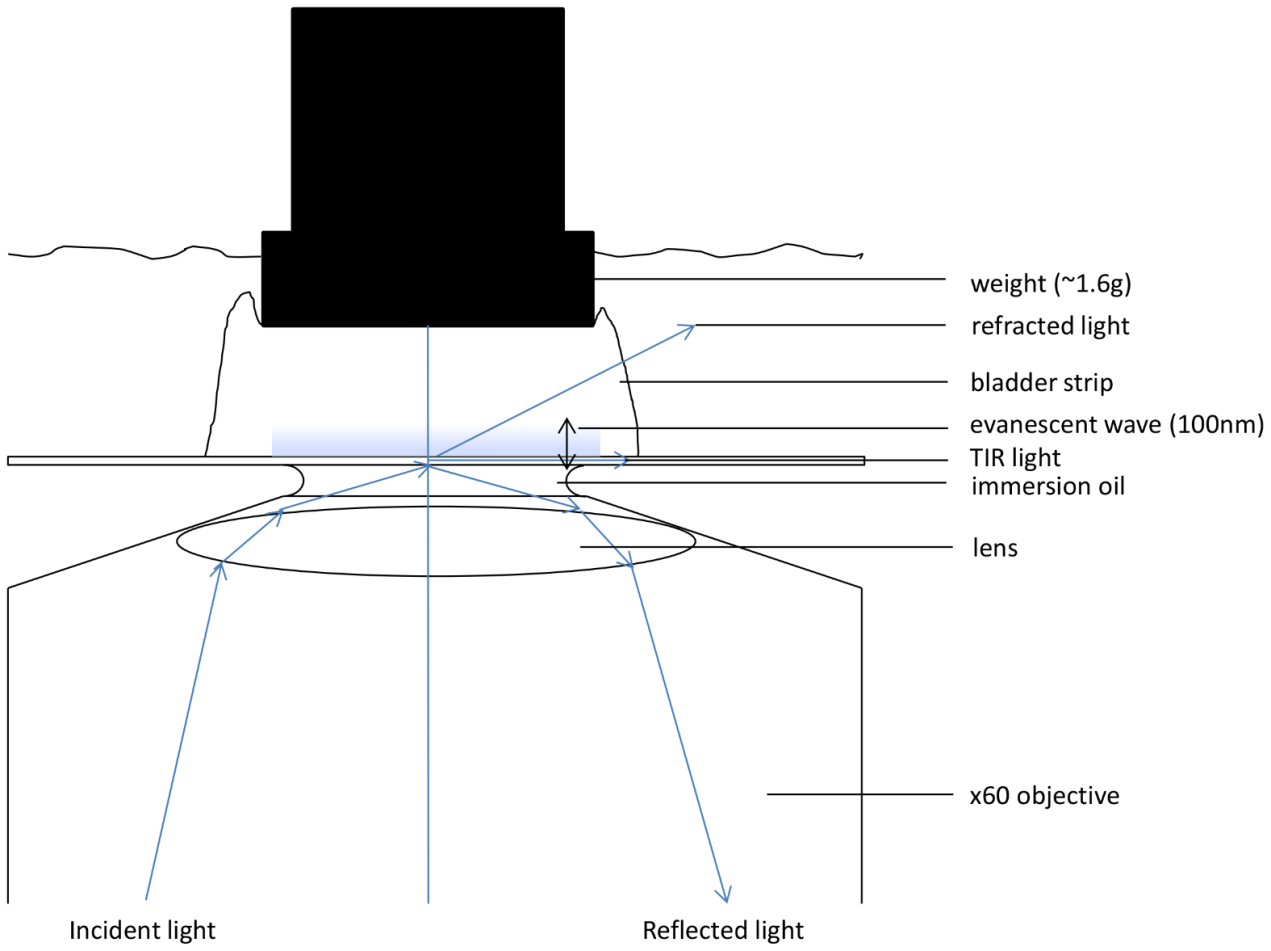
Schematic diagram depicting the TIRF microscopy approach used for the investigation of Ca^2+^ sparklets in an intact mouse urinary bladder smooth muscle syncytium. A TIRF signal relies upon the occurrence of total internal reflection at the liquid-glass interface, and creates an exponentially reducing evanescent field of up to 100 nm in which high resolution signals may be observed. In order to ensure that the outermost DSM cells of a mouse UBSM strip could be observed within the TIRF zone, a weight (1.6 g) was rested on top of the serosal side of the UBSM strip, allowing a TIRF image to be observed in UBSM cells. A typical variety of Ca^2+^ fluorescence signals including whole-cell Ca^2+^ transients (WCTs), Ca^2+^ waves and other sub-cellular Ca^2+^ events could be observed up to 2 h after placement of the weight, suggesting that such an approach was not particularly detrimental to the normal function of the UBSM strip. Not to scale.

Isolated strips of mouse urinary bladder strips were dissected as previously described. Following dissection, each strip was loaded with the fluorescent Ca^2+^ indicator Oregon Green BAPTA-1 AM (10 μM), dissolved in 1% DMSO –0.2% pluronic acid solution in oxygenated Krebs solution for 70 min at 35°C. Following indicator loading, the urinary bladder strip was placed, serosal side facing downwards, on the coverslip of a TIRF microscope (Nikon Instruments Eclipse-TI 2000 U, Tokyo, Japan) equipped with a 488 nm excitation laser and a CFI Plan Apo 60x/1.49na TIRF microscopy objective (Nikon Instruments, Tokyo, Japan). The UBSM was perfused with oxygenated Krebs solution at 25°C, and held in place using a small plastic-coated weight of approximately 1.6–1.7 g. Using the weight ensured that a signal could be detected within the TIRF zone (*i*.*e*. the evanescent field), whilst maintaining the position of the UBSM strip inside the perfusion chamber (Figure 1).

The smooth muscle strips were allowed to equilibrate for 10 min prior to imaging. Regions of the UBSM strip were selected for imaging, based upon; defined smooth muscle morphology; the occurrence of at least one Ca^2+^ sparklet during inspection; and a sufficiently stable TIRF signal. For each TIRF recording, a series of 2000 TIRF images were captured at a frame rate of 150 Hz, using an Ixon Ultra EMCCD fast imaging camera (Andor Technology plc, Belfast, UK). In order to minimize photobleaching, a maximum of 4 image sequences were recorded per smooth muscle strip, and the recordings were separated by a minimum period of 5 min. Where pharmacological treatments were applied, a minimum exposure period of 15 prior to imaging was used. Ca^2+^ signals could be detected more than 1 h after placement of the strip on the coverslip, implying that the setup was not detrimental to the survival of the UBSM strips. Example recordings of UBSM Ca^2+^ sparklets can be found in videos S1–S2.

### Image analysis

Image sequences were recorded using NIS-Elements imaging software (Nikon Instruments, Tokyo, Japan), saved as AVI files, and transferred to imageJ (version 1.47q) for offline analysis. Arbitrary selection of individual regions of interest (ROIs) for analysis can be time consuming, and heavily prone to investigator bias; therefore, the “LC pro” Ca^2+^ image analysis plugin for imageJ was used (available for download from http://imagej.nih.gov/ij/plugins/lc-pro/index.html; [14]. In order to detect Ca^2+^ sparklets, which are small, transient Ca^2+^ signals, an ROI size of 8 pixels was selected, thus excluding many larger Ca^2+^ transients from analysis. The LC pro image analysis program detects a ROI when a significant change in baseline Ca^2+^ concentration has occurred, and generates information for each ROI in the form of F/F_0_ values. The data for each ROI, in which at least one significant Ca^2+^ signal occurred, were analysed for the presence of Ca^2+^ sparklets using Labchart Pro 7 software (ADInstruments Japan, Okazaki, Japan). In order to be classified as a Ca^2+^ sparklet, an event had to be a minimum of 3 frames in duration, be 2.5 standard deviations above the baseline, and have a peak signal of at least 0.05 F/F_0_. Events that occurred simultaneously (or near simultaneously) in separate ROIs were excluded from the analysis, as detection in more than one ROI implies an event that is too large to be considered a sparklet. The occurrences of Ca^2+^ sparklets in the regions suggested by automated analysis were confirmed by visual inspection. Sparklet frequency data for each individual cell were corrected for cell membrane area because of the significant variations in the area of the cell membrane present within the TIRF zone.

### Drugs and chemicals

Atropine, α,β-methylene-adenosine 5′-triphosphate (α,β-meATP) and diltiazem were dissolved in Krebs solution and stored as either 1 or 10 mM stock solutions at −20°C until dilution on the day of use. R-(+)-Bay K 8644, cyclopiazonic acid (CPA), EGTA-AM and ML-9 were all dissolved in DMSO and stored as either 1 or 10 mM stock solutions at −20°C, until dilution as required. All drugs were purchased from Sigma-Aldrich Japan (Tokyo, Japan) except for Oregon Green 488 BAPTA-1 AM, EGTA-AM (Life Technologies, Tokyo, Japan) and R-(+)-Bay K 8644 (Tocris Biosciences, Bristol, UK).

### Statistical analysis

Data were presented and analysed using Graphpad 6.0 statistical analysis software or Origin 6.0 statistical analysis software. For comparisons of sparklet frequency, Wilcoxon's matched-pairs signed rank test was used on the basis that the data were paired and not normally distributed around the mean value. For comparisons of sparklet amplitudes, an unpaired Student's *t*-test was used on the basis that cells with no sparklets (and therefore a sparklet amplitude of 0) were removed from the analysis. In the case of EGTA-AM, sparklet frequencies were compared using a Mann-Whitney test, and amplitudes were compared using an unpaired Student's *t*-test. *P* values of less than 0.05 were considered statistically significant. For testing correlations between different paired datasets, the Spearman's rank correlation coefficient was used. *P* values of less than 0.05 were taken to indicate that sparklet frequencies were statistically dependent.

## Results

**Figure 2:**
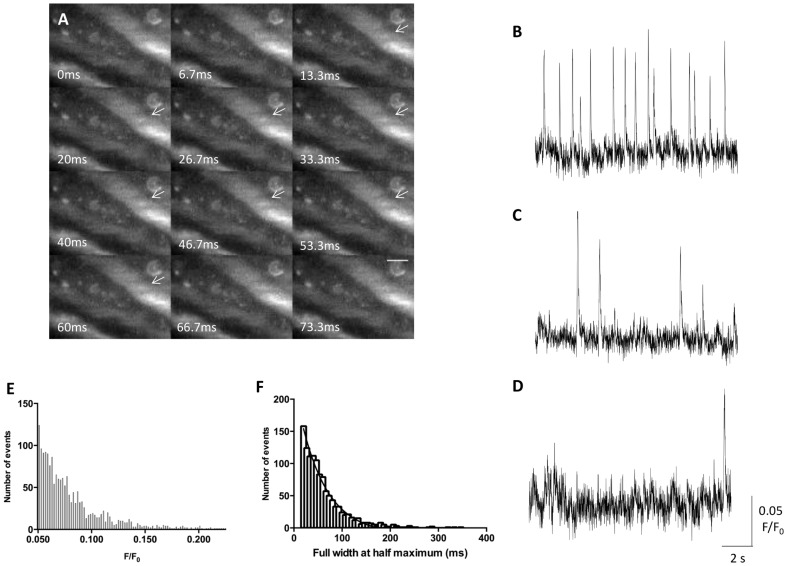
Characteristics of Ca^2+^ sparklets in DSM cells. A typical Ca^2+^ sparklet resulted in a transient increase in Ca^2+^ fluorescence, within an area of membrane approximately 50–60 μm^2^, that did not propagate in any direction, as indicated by the white arrows (A); scale bar 10 μm Ca^2+^ sparklets could be observed in various regions of the smooth muscle cell membrane, and were often, but not always, repeated at least once within the 13.3 s recording period (B–D). Amplitude distribution plot for Ca^2+^ sparklets recorded under control conditions, *i*.*e*. 1.8 mM Ca^2+^ and the absence of any agonists/antagonists (E). The duration of Ca^2+^ sparklets recorded under control conditions (F); events less than 3 frames in width were rejected from the analysis.

Ca^2+^ imaging of UBSM strips using an adapted TIRF microscopy approach revealed a population of small transient Ca^2+^ signals that occurred on or near the cell membrane of UBSM cells (Figure 2A). The observed events often, but not always, repetitively occurred at the same sites (Figures 2B–D). Under control conditions, these small, transient Ca^2+^ signals were detected in 47% (60/128) of preparations, and within the active preparations, in 72% (184/255) of cells. The detection of these transient Ca^2+^ signals (henceforth referred to as Ca^2+^ sparklets) in a relatively low percentage of preparations most likely reflected technical difficulties, *e*.*g*. excess connective tissues blocking access to the UBSM cell membranes, rather than an absence of activity. Over the course of the investigation, these signals were detected at minimum amplitude of 0.05 F/F_0_ and maximum amplitude of 0.27 F/F_0_ (Figure 2E).

### Ca^2+^ sparklets are cell membrane events

**Figure 3:**
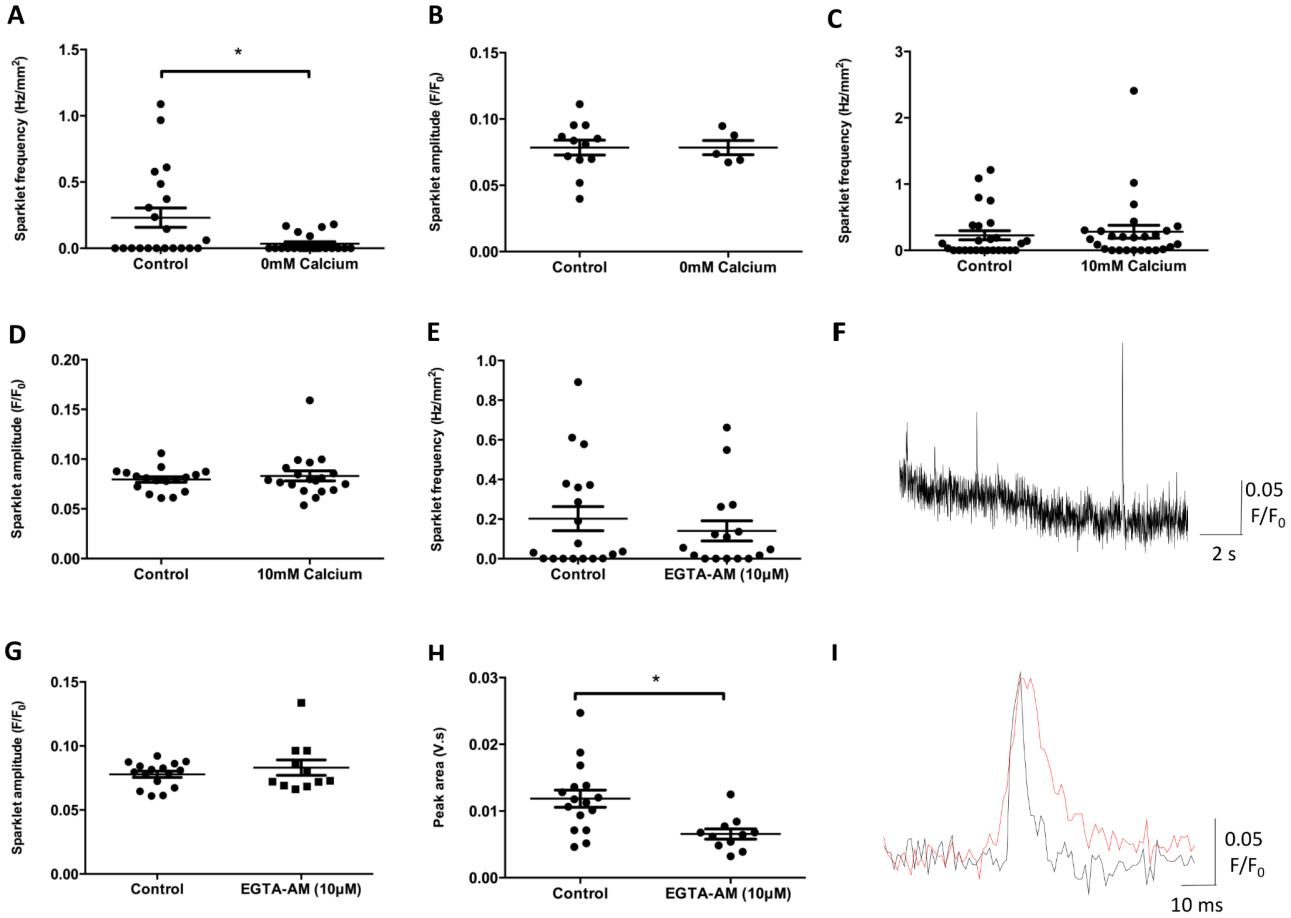
Ca^2+^ sparklets in DSM cells are confined to the cell membrane. Removal of extracellular Ca^2+^ significantly reduced the Ca^2+^ sparklet frequency without affecting the amplitude of the few remaining sparklets (A–B). In the presence of 10 mM external Ca^2+^, the amplitude and frequency of the Ca^2+^ sparklets remained largely unaffected, compared with controls (C–D). When UBSM strips were co-loaded with the Ca^2+^ chelator EGTA-AM (10 μM) together with the Ca^2+^ indicator (Oregon Green BAPTA-1-AM), Ca^2+^ sparklets could still be detected in the UBSM cells (E–F). Compared with controls, Ca^2+^ sparklet amplitude remained unaffected by the presence of EGTA-AM (G), whereas Ca^2+^ sparklet peak area was significantly reduced by the presence of EGTA-AM (H–I). The red plot denotes a sparklet under control conditions, and the black plot a sparklet in the presence of EGTA-AM (I). Note that when EGTA-AM was used, it was not possible to use paired controls, for the purpose of statistical comparisons; instead, the “controls” comprise 4 randomly selected control experiments from other datasets. Unpaired statistics were used for statistical comparisons of the data presented in panels E, G and H. An asterisk indicates a *P* value that is considered statistically significant (*P*<0.05, Wilcoxon matched-pairs signed rank test in A, Student's *t*-test in H).

The occurrence of Ca^2+^ sparklets in DSM cells was dependent upon the presence of Ca^2+^ in the extracellular Krebs solution. When the extracellular Ca^2+^ concentration was reduced to 0 mM for a 10 min period, Ca^2+^ sparklets were virtually abolished (Figure 3A; control: 0.23±0.07 Hz/mm^2^, 0 mM Ca^2+^: 0.035±0.01 Hz/mm^2^; n_c_ = 21 n_p_ = 5, *P*<0.05, Wilcoxon matched-pairs signed rank test). The mean amplitude of the sparklets observed in the absence of extracellular Ca^2+^ was not significantly different from that under control conditions (Figure 3B; control: 0.09±0.008 F/F_0_, 0 mM Ca^2+^: 0.08±0.005 F/F_0_; n_c_ = 12 for controls, 5 for 0 mM Ca^2+^, n_p_ = 5; *P*≥0.05, unpaired *t*-test,). In the presence of 10 mM Ca^2+^, sparklet frequency was not significantly different from control conditions (Figure 3C; control: 0.23±0.08 Hz/mm^2^, 10 mM Ca^2+^: 0.28±0.1 Hz/mm^2^; n_c_ = 23 n_p_ = 5; *P*≥0.05, Wilcoxon matched pairs signed rank test). Surprisingly, in spite of an increased extracellular Ca^2+^ gradient, the mean sparklet amplitude was unaffected by the presence of 10 mM Ca^2+^ (Figure 3D; control: 0.08±0.003 F/F_0_, 10 mM Ca^2+^: 0.08±0.006 F/F_0_; n_c_ = 17 for controls, 19 for 10 mM Ca^2+^, n_p_ = 5; *P*≥0.05, unpaired *t*-test).

In order to confirm that Ca^2+^ sparklets occurred at the cell membrane and were entirely independent of CICR, UBSM strips were co-loaded with the Ca^2+^ chelator EGTA, in the acetoxymethyl ester (AM) form, in order to chelate intracellular Ca^2+^. As a relatively slow Ca^2+^ chelator [15], non-fluorescent EGTA eliminates slow intracellular Ca^2+^ events that originate from the SR, leaving rapid Ca^2+^ entry from outside the cell unaffected [16]. Ca^2+^ sparklets were detected in the presence of EGTA (10 mM) at a similar frequency to that in unmatched controls (Figures 3E–F; control: 0.20±0.06 Hz/mm^2^, 10 mM EGTA-AM: 0.14±0.05 Hz/mm^2^; n_c_ = 19 cells for control, 16 for 10 mM EGTA-AM, n_p_ = 4; *P*≥0.05, Mann-Whitney test). No significant change in mean sparklet amplitude was detected in the presence of EGTA-AM (Figure 3G; control: 0.08±0.002 F/F_0_, 10 mM EGTA-AM: 0.08±0.006 F/F_0_; n_c_ = 16 cells for control, 11 for 10 mM EGTA-AM, n_p_ = 4, *P*≥0.05, Student's *t*-test), compared with controls. In the presence of EGTA-AM, there was a significant reduction in sparklet area, compared with controls (Figures 3H–I, control: 0.01±0.001 v.s, 10 mM EGTA-AM: 0.007±0.0008 v.s, n_c_ = 16 cells for control, 11 for 10 mM EGTA-AM, n_p_ = 4; *P*≤0.05, Student's *t*-test) thus demonstrating the chelating effects of EGTA. No Ca^2+^_sr_ mediated events (*i*.*e*. Ca^2+^ puffs, sparks, or waves) could be detected in the presence of EGTA-AM (unquantified observation).

### Ca^2+^ sparklets are mediated by VGCCs

**Figure 4:**
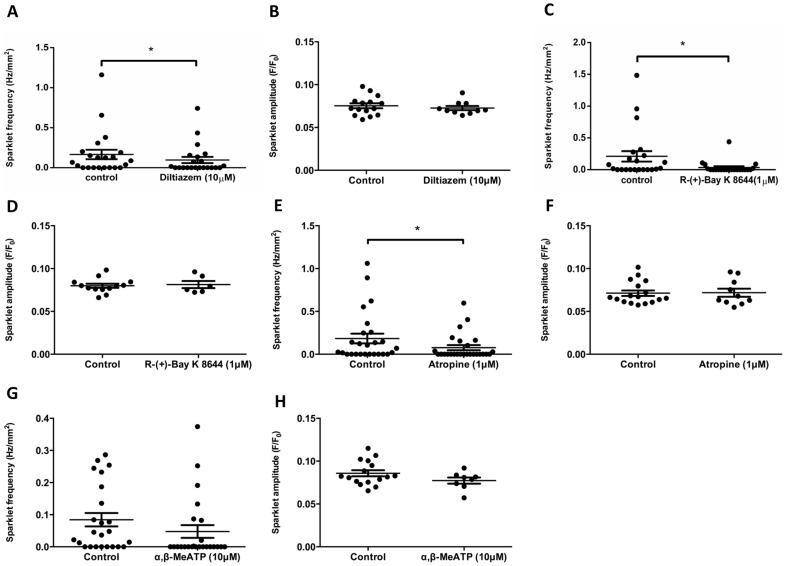
Ca^2+^ sparklets in DSM are mediated by VGCCs. Application of either R-(+)-Bay K 8644 (1 μM) or diltiazem (10 μM) significantly reduced the frequency of the Ca^2+^ sparklets whilst leaving the amplitudes of the remaining sparklets largely unaffected (A–D). Atropine (1 μM) significantly reduced the frequency of Ca^2+^ sparklets without affecting the amplitude of the remaining sparklets (E–F). α,β-meATP (10 μM) had no significant effects upon either amplitude or frequency of the Ca^2+^ sparklets (G–H). An asterisk indicates a *P* value that is considered statistically significant (*P*<0.05, Wilcoxon matched-pairs signed rank test).

Having demonstrated that Ca^2+^ sparklets are cell membrane events, they were investigated pharmacologically to identify potential channels that may be involved in their generation. Ca^2+^ sparklet frequency, was significantly reduced by the L-type Ca^2+^ channel blockers, diltiazem (Figure 4A; control: 0.17±0.06 Hz/mm^2^, 10 μM diltiazem: 0.097±0.04 Hz/mm^2^; n_c_ = 22, n_p_ = 5; *P*<0.05, Wilcoxon matched pairs signed rank test) and R-(+)-Bay K 8644 (Figure 4C; control: 0.2±0.08 Hz/mm^2^, 1 μM R-(+)-Bay K 8644 (1 μM): 0.034±0.021 Hz/mm^2^, n_c_ = 22 n_p_ = 5; *P*<0.05, Wilcoxon matched pairs signed rank test). The amplitudes of the sparklets remaining in the presence of both blockers were not significantly different from those of the controls (Figure 4B; control: 0.08±0.003 F/F_0_, 10 μM diltiazem: 0.07±0.002 F/F_0_; n_c_ = 15 for control, 10 for 10 μM diltiazem, n_p_ = 5; *P*≥0.05, unpaired *t*-test: Figure 4D, control: 0.08±0.002 F/F_0_, 1 μM R-(+)-Bay K 8644 (0.08±0.004 F/F_0_, n_c_ = 13 for control, 6 for 1 μM R-(+)-Bay K 8644, n_p_ = 5, *P*≥0.05, unpaired *t*-test).

UBSM strips undergo small spontaneous depolarizations that have been shown to be largely purinergic in origin, and can lead to spontaneous contractions through activation of VGCCs [17],[18]. In order to determine the effects of spontaneous neurotransmitter release upon VGCC-mediated Ca^2+^ sparklets, α,β-meATP or atropine were applied to UBSM strips. In the presence of atropine (1 μM), there was a significant decrease in sparklet frequency (Figure 4E control: 0.18±0.06 Hz/mm^2^, 1 μM atropine: 0.078±0.03 Hz/mm^2^; n_c_ = 26, n_p_ = 5, *P* = 0.0483, Wilcoxon matched-pairs signed rank test) but not amplitude (Figure 4F, 1 μM atropine: 0.07±0.003 F/F_0_, control: 0.07±0.005 F/F_0_; n_c_ = 17 for control, 10 for atropine, n_p_ = 5; *P*≥0.05, unpaired *t*-test), compared with the controls. In the presence of 10 μM α,β-meATP, there was no statistically significant difference in sparklet frequency (Figure 4G; control: 0.084±0.02 Hz/mm^2^, 10 μM α,β-meATP: 0.048±0.02 Hz/mm^2^; n_c_ = 24, n_p_ = 5, *P* = 0.0507, Wilcoxon matched-pairs signed rank test) or amplitude (Figure 4H; control: 0.09±0.004 F/F_0_, 10 μM α,β-meATP: 0.08±0.004 F/F_0_; n_c_ = 16 for control, 8 for 10 μM α,β-meATP, n_p_ = 5, *P*≥0.05, unpaired *t*-test), compared with the controls.

### Ca^2+^ sparklets are protein kinase C (PKC) dependent

**Figure 5:**
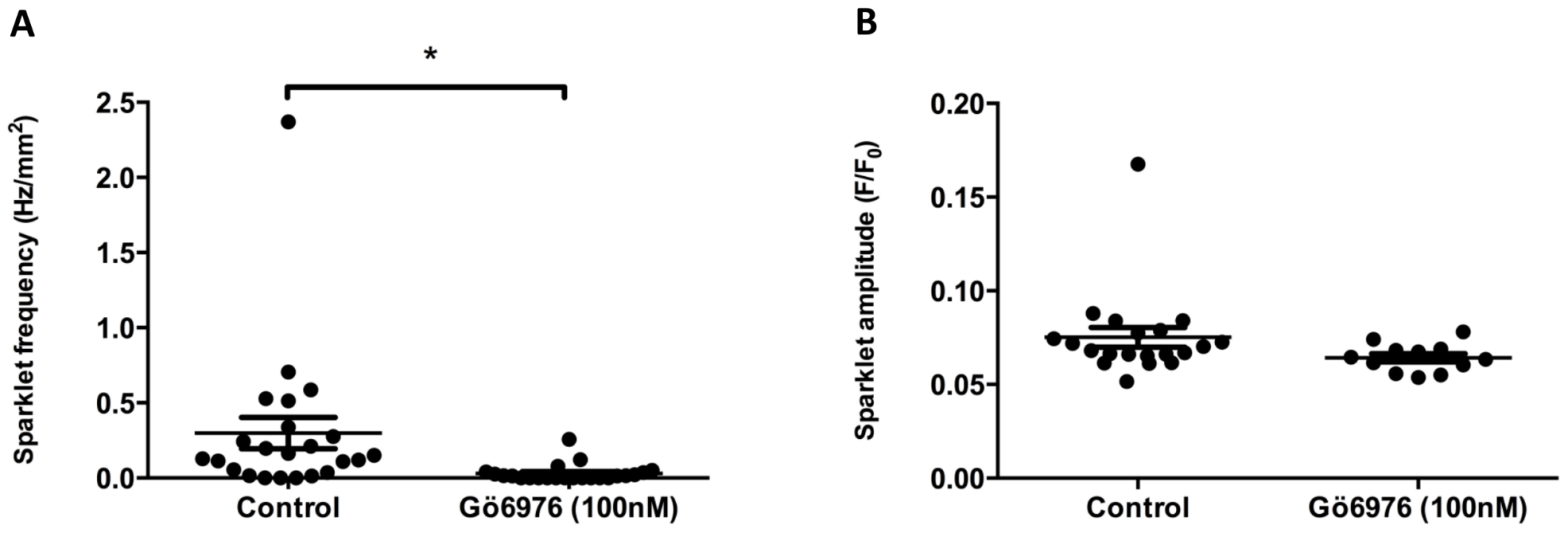
Ca^2+^ sparklets in DSM are PKC-dependent. Application of the PKC inhibitor Gö6976 (100 nM) significantly reduced the frequency of Ca^2+^ sparklets (A) whilst leaving the amplitudes of the remaining sparklets largely unaffected (B). An asterisk indicates a *P* value that is considered statistically significant (*P*<0.05, Wilcoxon matched-pairs signed rank test).

Several investigations in vascular smooth muscle indicate that Ca^2+^ sparklets require PKC-mediated phosphorylation of VGCCs. When UBSM strips were incubated in the presence of Gö6976 (100 nM), a PKC inhibitor, there was a significant decrease in sparklet frequency (Figure 5A; control: 0.30±0.1 Hz/mm^2^, 100 nM Gö6976: 0.030±0.01 Hz/mm^2^, n_c_ = 23, n_p_ = 5; *P*≤0.05, Wilcoxon matched-pairs signed rank test) but not amplitude (Figure 5B, control: 0.08±0.005 F/F_0_, Gö6976: 0.06±0.002 F/F_0_, n_c_ = 20 for controls, 12 for Gö6976, n_p_ = 5, *P*≥0.05, unpaired *t*-test).

### Ca^2+^ sparklets are affected by signals from the SR

**Figure 6:**
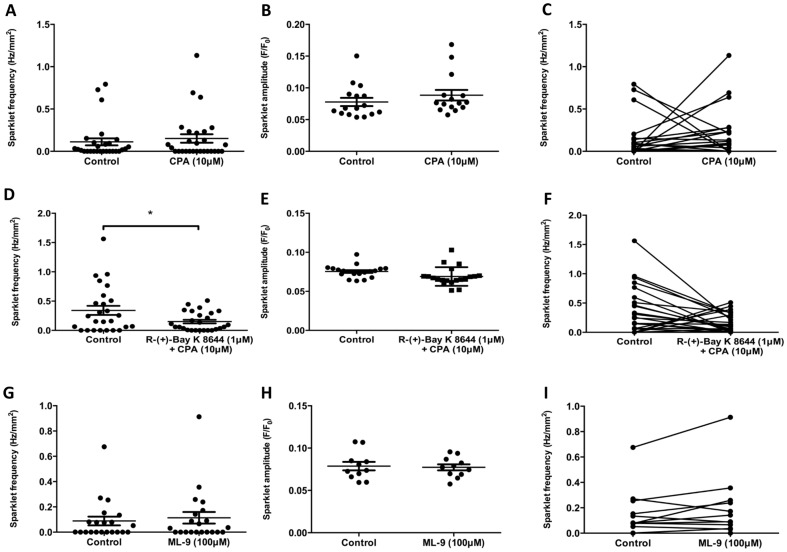
Ca^2+^ sparklets in DSM are affected by, but not dependent upon, signals from the SR. In the presence of CPA (10 μM), the overall sparklet frequency (A) and amplitude (B) were not significantly different from those in controls. The sparklet frequencies of specific cells were significantly altered by exposure to CPA (10 μM) to the point where sparklet frequencies before and after application of CPA were found not to be statistically dependent (C). In the presence of the VGCC antagonist R-(+)-Bay K 8644 (1 μM) and CPA (10 μM), there was a significant decrease in the frequency (D) but not amplitude (E) of Ca^2+^ sparklets. Sparklet frequencies were once again found not to be statistically dependent (F). ML-9 (100 μM) was used to inhibit interactions between STIM1 and the cell membrane. In the presence of ML-9 (100 μM), there were no significant changes in sparklet frequency (G) or amplitude (H) compared with the controls, and sparklet frequencies were found to be statistically dependent. An asterisk indicates a *P* value that is considered statistically significant (*P*<0.05, Wilcoxon matched-pairs signed rank test).

**Figure 7:**
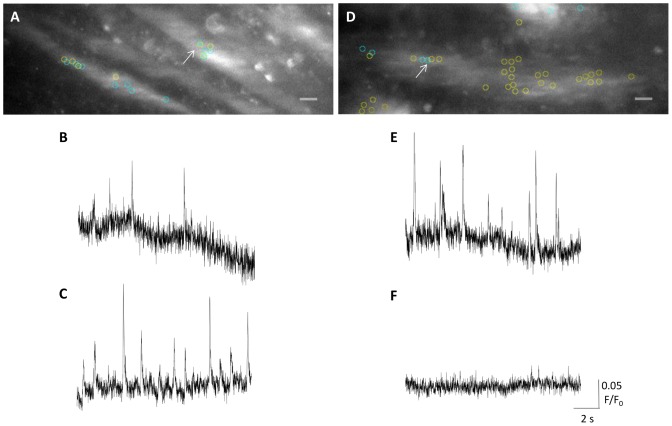
Ca^2+^ sparklets in DSM occur at different frequencies in the presence of CPA (10 μM). Overlaid images (x60) of a preparation where (A) CPA (10 μM) caused an increase in sparklet frequency, and (D) where CPA (10 μM) caused a decrease in sparklet frequency, compared with the controls. Yellow circles indicate sites at which at least one sparklet could be detected under control condition; light blue circles indicate sites where at least one sparklet could be detected in the presence of CPA (10 μM). In image A, the arrow refers to the site from which B (control) and C (10 μM CPA) were recorded. It appears that CPA (10 μM) can generate increased sparklet frequencies by acting upon the same or similar membrane locations. In image D, the arrow refers to the site from which E (control) and F (10 μM CPA) were recorded. It was difficult to detect any sparklet sites in the presence of CPA (10 μM) in image D. Images are overlaid with 50% opacity; small variations in focus and/or small amounts of movement may be responsible for the blurred appearances of the images. The scale bar, (10 μm) applies to all traces.

In order to investigate the role of the SR in the generation of Ca^2+^ sparklets, CPA (10 μM) was used to deplete SR Ca^2+^ stores, active depletion of SR Ca^2+^ stores favours the occurrence of SOCE. In the presence of CPA, there was no statistically significant difference in the overall frequency (Figure 6A; control: 0.11 ± 0.04 Hz/mm^2^, 10 μM CPA: 0.15 ± 0.05 Hz/mm^2^; n_c_ = 28, n_p_ = 5; *P*≥0.05, Wilcoxon matched-pairs signed rank test) or amplitude (Figure 6B; control: 0.08±0.06 F/F_0_, 10 μM CPA: 0.09±0.008 F/F_0_; n_c_ = 16, n_p_ = 5; *P*≥0.05, unpaired *t*-test) of Ca^2+^ sparklets, compared with the controls. Although no significant change in mean sparklet frequency was detected following the addition of 10 μM CPA, the statistical correlation of events before and after the addition of CPA differed significantly to that observed for the other pharmacological interventions. Cells with a higher sparklet frequency under control conditions, generally showed a reduction in sparklet frequency in the presence of 10 μM CPA, whilst other cells saw an increase in sparklet frequency, typically from a very low frequency under control conditions (Figure 7). Correlation of sparklet frequency was investigated using Spearman's rank correlation coefficient. Sparklet frequency under control conditions was found to be statistically independent of sparklet frequency in the presence of 10 μM CPA (Figure 6C; Spearman's rank correlation, rs = 0.2, *P*≥0.05), indicating that a high sparklet frequency for any given cell under control conditions is in no way correlated with a high sparklet frequency in the presence of 10 μM CPA. In all other datasets using other pharmacological interventions, sparklet frequencies before and after the intervention were found to be statistically dependent on each other; this demonstrates a statistically significant change in the locations of Ca^2+^ sparklets, but not in the overall mean sparklet frequency (Table S1). CPA-induced events appeared very similar, in terms of size, shape and mean overall frequency, to the VGCC-mediated sparklets detected under control conditions. In order to investigate the channels involved in CPA induced sparklet activity, UBSM strips were exposed to 10 μM CPA in the presence of 1 μM R-(+)-Bay K 8644. Combined application of CPA and R-(+)-Bay K 8644 resulted in a significant decrease in Ca^2+^ sparklet frequency (Figure 6D; control: 0.34 ± 0.08 Hz/mm^2^, 1 μM R-(+)-Bay K 8644+10 μM CPA: 0.15 ± 0.03 Hz/mm^2^; n_c_ = 26, n_p_ = 5, *P*<0.05, Wilcoxon matched-pairs signed rank test) but not amplitude (Figure 6E; control: 0.08±0.002 F/F_0_, 1 μM R-(+)-Bay K 8644+10 μM CPA: 0.07 ± 0.003 F/F_0_, n_c_ = 21 for control, 22 for 1 μM R-(+)-Bay K 8644+10 μM CPA, n_p_ = 5, *P*≥0.05, unpaired *t*-test), compared with the controls. When correlations between datasets were compared, the data were found not to be statistically dependent (Figure 6F, Spearman's rank correlation, rs = 0.1, *P*≥0.05). In order to confirm that Ca^2+^ sparklets in the absence of CPA were not mediated by stromal interacting molecule-1 (STIM1), and therefore probably caused by passive depletion of Ca^2+^_sr_, ML-9 (100 μM), an inhibitor of STIM1 plasma membrane interactions, was applied to UBSM strips. In the presence of 100 μM ML-9, there was no statistically significant change in overall sparklet frequency (Figure 6G; control: 0.088 ± 0.03 Hz/mm^2^, 100 μM ML-9: 0.11±0.05 Hz/mm^2^; n_c_ = 23, n_p_ = 5, *P*<0.05, Wilcoxon matched pairs signed rank test) or amplitude (Figure 6H; control: 0.08±0.02 F/F_0_, 100 μM ML-9: 0.08±0.01 F/F_0_; n_c_ = 11, n_p_ = 5, *P*≥0.05, unpaired *t*-test), compared with the controls.

## Discussion

### Ca^2+^ sparklets are restricted to the cell membrane, and require VGCCs for activation

The small, discrete Ca^2+^ elevations detected in this investigation were limited to the cell membranes of UBSM cells. There are several pieces of evidence that support this claim: Firstly, signals of this nature cannot be routinely observed with high magnification epifluorescence microscopy or confocal laser scanning microscopy, implying that the superior resolution, and restricted excitation zone of a TIRF microscope was required to image the observed events. Secondly, the removal of extracellular Ca^2+^ significantly reduced the frequency of the Ca^2+^ sparklets, indicating that the source of Ca^2+^ for these events is extracellular (Figures 3A–B). The most likely explanation for the occurrence of a limited number of events in the presence of 0 mM Ca^2+^ is the trapping of some residual Ca^2+^ containing solution between the coverslip and the UBSM cell membrane, very close proximity between the specimen and coverslip being a requirement of TIRF imaging. When UBSM strips were co-loaded with non-fluorescent EGTA-AM at similar concentrations to the Ca^2+^ indicator, Ca^2+^ sparklets could still be detected, with a significant reduction in peak area but not amplitude (Figures 3G–I). Because EGTA is a slow Ca^2+^ chelator [1],[15], no fluctuations in intracellular Ca^2+^ concentrations were detected that originate from the SR or indeed other intracellular organelles (unquantified observation).

In the presence of the VGCC inhibitors R-(+)-Bay K 8644 or diltiazem, there was a significant reduction in the frequency of Ca^2+^ sparklets (Figures 4A–D), indicating that Ca^2+^ sparklets are VGCC-mediated. These findings are in agreement with previous reports of small, non-voltage dependant openings of VGCCs detected in isolated vascular smooth muscle cells [10],[11],[12],[13],[19], although some debate continues about the occurrence and physiological importance of these events [20].

The main parasympathetic neurotransmitters in UBSM are acetylcholine and ATP, and it has been shown previously that M3 muscarinic [21] and P2X_1_ [22] receptors, respectively, are the major receptor subtypes. In the presence of atropine, there was a significant decrease in Ca^2+^ sparklet frequency but not amplitude (Figures 4E–F). Given that muscarinic receptors are G protein-coupled and typically not associated with rapid Ca^2+^ entry from the external environment, this is a somewhat surprising finding. It is suggested given the PKC-dependent nature of Ca^2+^ sparklets (Figures 5A–B) that the inhibition of Ca^2+^ sparklet frequency by atropine was the result of a reduction in M3 receptor activation, by spontaneously released acetylcholine. Under control conditions, spontaneous acetylcholine release may promote the occurrence of Ca^2+^ sparklets by producing a basal level of stimulation of the G_q/11_ downstream signalling cascade, which in turn will activate PKC. Investigations of similar events in isolated vascular smooth muscle cells indicate that these events are likely to be PKC-dependent [10],[11].

In the presence of α,β-meATP, there appeared to be a trend towards a reduction in Ca^2+^ sparklet frequency, although it is important to note that statistical significance was not reached. Electrophysiological studies using this concentration of α,β-meATP at 10 μM typically show that P2X_1_ receptor-mediated events are abolished [18]. Therefore, the statistically insignificant, apparent downward trend in the Ca^2+^ sparklet frequency is also likely to be indirect. It is suggested that this effect may be due to the loss of smooth muscle activation and resultant Ca^2+^ mobilisation followed by some extrusion of intracellular Ca^2+^ that would normally occur in response to spontaneously released ATP.

### Are Ca^2+^ sparklets single channel events?

In order to determine whether these events arise as a result of openings of a single ion channel/complex of ion channels or the near synchronous opening of several channels, a defined set of criteria need to be considered [23],[24]: (1) the recording volume should ideally be very small (<1 fL); (2) events should be quantal; (3) amplitude steps should depend upon the Ca^2+^ electrochemical gradient; (4) the amplitude steps should be largely unaffected by the concentration or nature of the agonist used; (5) the durations of the events should be exponentially distributed; and (6) the channels should be highly Ca^2+^ permeable, and have an identified single channel conductance.

Mouse UBSM strips contain smooth muscle cells that can be up to 500 μm in length, therefore, allowing for some electrical coupling, the volume of each compartment is almost certainly greater than 1fL (criterion 1). It was also not possible to plot a multiple Gaussian distribution of event amplitude (criterion 2), and the size of the events does not appear to be dependent upon the Ca^2+^ electrochemical gradient (criterion 3), to the point that increasing the extracellular Ca^2+^ concentration to 10 mM did not result in a statistically significant increase in the amplitude of the events (Figure 3D). In the presence of 0 mM external Ca^2+^, the overall number of events was reduced to almost zero; however, among the very rare events that were detected, there was no obvious change in amplitude.

In a physiological context, it may be very difficult to satisfy criterion 2 by plotting a multiple Gaussian function, due to interference from criterion 3. Smooth muscle cells in a poorly coupled electrical syncytium rarely maintain an entirely constant voltage in the same way as voltage-clamped isolated myocytes. Indeed, the resting membrane potential (RMP) can reportedly vary between −35 and −60 mV [17] or −35.9 and −46.9 mV in mouse UBSM strips [25]. If the events monitored in this investigation are dependent upon electrochemical gradient (criterion 3), it is unlikely that a good Gaussian fit (criterion 2) can be plotted, given that the RMP differs significantly between UBSM cells.

Distinct amplitude steps are difficult to identify in the model used. However, there was no statistically significant change in the mean amplitudes of the events in response to any of the pharmacological treatments used in the present investigation (criterion 4; Figures 4B, 4D, 4F, 4H, 5B, 5E and 6B). The duration of the individual events detected in this investigation appeared to be exponentially distributed (Figure 1F, criterion 5). VGCCs have been shown elsewhere to have detectable single channel Ca^2+^ conductance in isolated vascular smooth muscle cells (criterion 6) [10]. These findings indicate that the events observed may be single channel events, although conclusively answering this question using this model may not be possible due to the limitations of current technologies.

### Ca^2+^ sparklets are dependent upon PKC activation

Where UBSM strips were incubated in the presence of the PKC inhibitor, Gö6976, there was a significant reduction in Ca^2+^ sparklet frequency, but once again, not detectable change in amplitude (Figures 5A–B). These data strongly suggest that, as observed elsewhere [10],[11], Ca^2+^ sparklets require PKC activity. Whether basal PKC activity alone is sufficient for frequent Ca^2+^ sparklets, or if an unknown PKC-promoting signal is responsible for these events, remains unclear at the present time.

### Ca^2+^ sparklets are affected by signals from the SR

In the presence of CPA, there was no noticeable effect upon the overall mean frequency of Ca^2+^ sparklets in UBSM (Figure 6A). However, it became apparent that UBSM cells with a higher sparklet frequency under control conditions showed a significant reduction in frequency in the presence of CPA. At the same time, a number of cells in which Ca^2+^ sparklets could either not, or barely, be detected under control conditions showed a significant increase in sparklet frequency (Figures 6C and 7). No significant correlation was detected between the paired sparklet frequencies in the absence and presence of CPA, whereas paired sparklet frequencies in response to other interventions were always statistically correlated (Table S1).

This finding suggests that inhibition of SERCA, and the resultant depletion of intracellular Ca^2+^ stores, acts as a dual regulator of Ca^2+^ sparklets. Depletion of SR Ca^2+^ stores, using either; CPA, thapsigargin or ryanodine in a wide range of non-excitable and excitable cell types has been shown elsewhere to activate a non-stochastic form of Ca^2+^ entry that is widely reported to be mediated by STIM1/ORAI and is referred to as SOCE [9]. It has recently been demonstrated that, in addition to SOCE, store-inhibited Ca^2+^ entry (SIC) can occur through largely the same pathways as SOCE [26],[27]. Thus far, the major candidate for SIC, following co-assembly with activated STIM1/ORAI complexes, is the L-type Ca^2+^ channel [26],[27]. It is suggested in this investigation that SIC may in fact be occurring alongside SOCE, often in neighbouring cells, or that SOCE occurs through a different set of membrane proteins to Ca^2+^ sparklets under control conditions. Three key pieces of evidence indicate that the former, rather than the latter, situation is occurring.

First, in the presence of R-(+)-Bay K 8644, there was no significant activation of SOCE by CPA; indeed, there was a statistically significant reduction in the frequency of Ca^2+^ sparklets, indicating that CPA-induced events are VGCC-dependent (Figures 6D–F). Second, the amplitudes, detection conditions and other biophysical characteristics of the cell membrane events were largely indistinguishable, regardless of the presence of CPA or indeed any other agonist/antagonist (Figures 4B, 4D, 4F, 4H, 5B, 5E, 6B and 7). Third, in some cases, Ca^2+^ sparklets occurred in the same or very similar (allowing for small differences caused by slight variations in focus and movement of the preparation) membrane areas of the same cells in both the absence and presence of CPA, often at very different frequencies (Figure 7). This would imply that at least in some cases, the same membrane protein complexes are involved in spontaneous Ca^2+^ sparklets and CPA-induced events.

### What is the physiological role of VGCC sparklets?

In cardiac muscle, VGCC Ca^2+^ sparklets are directly coupled with ryanodine receptors on the SR, and activate excitation-contraction coupling (ECC) through local activation of 4–6 ryanodine receptors that can lead to a WCT [28],[29]. In vascular smooth muscle, VGCC sparklets are not functionally coupled with ryanodine receptors, but appear to contribute to Ca^2+^_cyt_ and indirectly Ca^2+^_sr_ [12]. Therefore, an increase in sparklet frequency may lead to increased smooth muscle excitability and ultimately hypertension [30]. In UBSM strips, Ca^2+^ sparklets can be detected using TIRF microscopy. These events can be affected by depletion of Ca^2+^_sr_, but can also occur independently of any signals from the SR, suggesting an additional role in local regulation of cell membrane processes.

Other reports indicate that voltage dependant VGCC activity is the major conduit for Ca^2+^ entry into smooth muscle [20],[31]. In rat vascular smooth muscle, it has been estimated that there are approximately 5000 VGCCs per cell, at a density of approximately 4 per μm^2^ [32]. Investigations using smooth muscle cells isolated from mouse urinary bladder indicate that VGCCs are not uniformly distributed across the cell membrane, but are distributed in clusters [33]. Taken together, these data would indicate that a very small subset of the total VGCC population of a UBSM cell are able to generate Ca^2+^ sparklets, although the clustered nature of VGCC distribution suggests that these events are due to activation of either single channels or complexes of channels.

Given that the indicator used in the present study chelates intracellular Ca^2+^, it seems plausible that the use of BAPTA-1-based Ca^2+^ indicators for the monitoring of VGCC sparklets may produce a passive depletion of intracellular Ca^2+^ stores, and resultant SOCE [34]. This would indicate that the experimental conditions used during this investigation are in fact causing or exaggerating the physiological effects reported herein. If this effect is indeed induced or exaggerated by the use of a BAPTA-1 based Ca^2+^ indicator, it seems reasonable to expect a significant increase in sparklet frequency in the presence of a second chelator, in this case EGTA-AM [16]. In this study, EGTA-AM did not significantly affect sparklet frequency (Figure 3E), again suggesting that under control conditions, Ca^2+^ sparklets are unrelated to Ca^2+^_sr_. The AM Ca^2+^ indicator loading protocol used in this investigation may have caused significant variations in the indicator concentration, resulting in variable concentrations of Ca^2+^_sr_ and Ca^2+^_cyt_, although in many cases SR Ca^2+^ stores were clearly not depleted, as evidenced by the occurrence of intracellular Ca^2+^ events, sometimes in the same cells as VGCC sparklets (Videos S3–S4). The finding that application of ML-9, an inhibitor of STIM1-cell membrane interactions, did not affect sparklet frequency (Figures 6G–I) confirms the view that Ca^2+^ sparklets under control conditions are not caused by passive depletion of Ca^2+^_sr_.

VGCC-dependent sparklets clearly represent a mechanism of capacitative Ca^2+^ entry in UBSM. However, under control conditions these events do not depend upon depletion of Ca^2+^_sr_, although they are affected by pharmacological depletion of Ca^2+^_sr_ (Figures 6A–F). Electrophysiological investigations indicate that in UBSM cells strips, only 38% of cells studied were able to generate VGCC mediated WCTs, all of which are neurogenic in origin, and therefore not dictated by the intracellular Ca^2+^ concentration of the UBSM cell [18]. This would indicate the need for an additional Ca^2+^ entry pathway in UBSM cells devoid of spontaneous VGCC activity.

In excitable cells, it is suggested that SOCE can occur through VGCCs, at present, evidence for this effect is limited to one report of thapsigargin induced currents in the non-excitable U937 cell line that are sensitive to dyhidropyridine antagonists [35]. These findings suggest that although Ca^2+^ can clearly enter UBSM cells through voltage-induced activation of VGCCs, this is not the only Ca^2+^ entry pathway, although the extent to which VGCC sparklets contribute to Ca^2+^ entry under physiological conditions is unclear.

## Conclusions

This study uses TIRF microscopy for the investigation of Ca^2+^ sparklets in urinary bladder smooth muscle tissue, and presents a novel method which potentially could be applied to experimental studies of other types of smooth muscle. The small, transient Ca^2+^ signals observed in the present study have been widely reported in isolated vascular smooth muscle cells; this investigation demonstrates the occurrence of these events in an intact electrical syncytium. Ca^2+^ sparklets under control conditions do not reflect depleted Ca^2+^_cyt_ or Ca^2+^_sr_. Pharmacological depletion of Ca^2+^_sr_ activates Ca^2+^ sparklets in cells with a low initial sparklet frequency, but inhibits Ca^2+^ sparklets in cells with a higher initial sparklet frequency, demonstrating that Ca^2+^ sparklets in UBSM are a type of SOCE. The effects of Ca^2+^_sr_ depletion are mediated by VGCC complexes, demonstrating a SOCE pathway that has not been reported previously in excitable cells, and suggesting that depletion of Ca^2+^_sr_ can both inhibit and activate VGCCs.

## Supporting Information

Table S1 **Results of Spearman's rank correlation analysis.** Paired data were tested for statistical dependence, using the null hypothesis that sparklet frequency data in the presence of each of the various agonists/antagonists were statistically independent of those in the controls.
(DOCX)

Video S1 **Recording of spontaneous Ca^2+^ sparklets from a DSM strip loaded with Ca^2+^ indicator.** Scale bar, 10 μm.
(AVI)

Video S2 **Recording of spontaneous Ca^2+^ sparklets in the presence of the VGCC antagonist, R-(+)-Bay K 8644 (1 μM).** This recording is paired with the recording shown in video S1. Scale bar, 10 μm.
(AVI)

Video S3 **A DSM strip loaded with Ca^2+^ indicator, showing various Ca^2+^ sparklets in addition to a WCT (ROI 1, frames 530–550) and a Ca^2+^ puff (ROI 2, frames 1800–1815).** Scale bar, 10 μm.
(AVI)

Video S4 **A DSM strip loaded with Ca^2+^ indicator, showing various intracellular Ca^2+^ waves.** The poorly defined edges and propagating nature of many of the signals suggest that an intracellular Ca^2+^ signal is occurring.
(AVI)
