# Supplementary material for: L-Type Ca2+ Channel Sparklets Revealed by TIRF Microscopy in Mouse Urinary Bladder Smooth Muscle
Source: PLoS One. 2014 Apr 3;9(4):e93803. doi: 10.1371/journal.pone.0093803 (PMC3974850; doi:10.1371/journal.pone.0093803)
Supplement: Table S1 — Results of Spearman's rank correlation analysis. Paired data were tested for statistical dependence, using the null hypothesis that sparklet frequency data in the presence of each of the various agonists/antagonists were statistically independent of those in the controls. (DOCX) [file pone.0093803.s001.docx]

**Table S1.**

| Treatment | Spearman's rank rs (one tailed) | *P* value | significant? |
| --- | --- | --- | --- |
| 0 mM Ca^2+^ | 0.51 | 0.009 | ** |
| 10 mM Ca^2+^ | 0.34 | 0.004 | * |
| EGTA-AM (10 mM) | Data not paired |  |  |
| R-(+)-Bay K 8644 (1 μM) | 0.69 | 0.0002 | *** |
| Diltiazem (10 μM) | 0.75 | <0.0001 | *** |
| Atropine (1 μM) | 0.37 | 0.03 | * |
| α,β-meATP (10 μM) | 0.59 | 0.001 | ** |
| CPA (10 μM) | 0.17 | 0.2 | ns |
| R-(+)-Bay K 8644 (1 μM) + CPA (10 μM) | 0.14 | 0.25 | ns |
| ML-9 (100 μM) | 0.98 | <0.0001 | *** |
| Gӧ6976 (100 nM) | 0.46 | 0.01 | * |
